# 5-Methyl-1,2-oxazole-3-carb­oxy­lic acid

**DOI:** 10.1107/S160053681103532X

**Published:** 2011-09-03

**Authors:** Mei-Ling Pan, Yang-Hui Luo, Jin-Feng Li

**Affiliations:** a Ordered Matter Science Research Center, College of Chemistry and Chemical Engineering, Southeast University, Nanjing 210096, People’s Republic of China

## Abstract

In the crystal structure of the title compound, C_5_H_5_NO_3_, all the non-H atoms are approximately coplanar: the carb­oxy O atoms deviating by 0.013 (2) and −0.075 (2) Å from the isoxazole ring plane. In the crystal, the molecules form inversion dimers linked by pairs of O—H⋯O hydrogen bonds and the dimers stack *via* π–π inter­actions [centroid–centroid distance = 3.234 (2) Å].

## Related literature

The title compound is a potent inhibitor of the monoamine oxidase enzyme and multidentate ligand for transition metals, see: Birk & Weihe (2009 ▶).
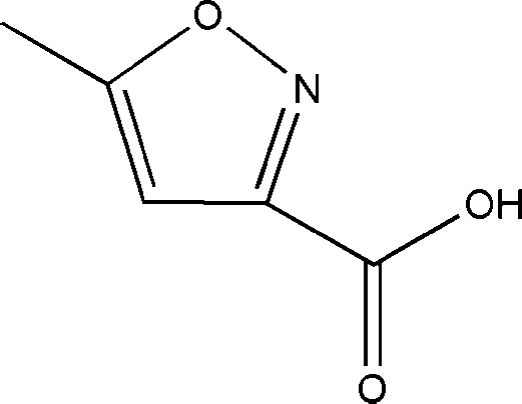


## Experimental

### 

#### Crystal data

C_5_H_5_NO_3_
*M* *_r_* = 127.10
Triclinic, 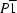

*a* = 4.9125 (10) Å
*b* = 5.6909 (11) Å
*c* = 10.464 (2) Å
α = 82.21 (3)°
β = 79.72 (3)°
γ = 78.96 (3)°
*V* = 280.96 (10) Å^3^
*Z* = 2
Mo *K*α radiation
μ = 0.13 mm^−1^
*T* = 293 K
0.20 × 0.20 × 0.20 mm

#### Data collection

Rigaku SCXmini diffractometer
Absorption correction: multi-scan (*CrystalClear*; Rigaku, 2005 ▶) *T*_min_ = 0.975, *T*_max_ = 0.975
2923 measured reflections
1283 independent reflections
1052 reflections with *I* > 2σ(*I*)
*R*_int_ = 0.079

#### Refinement

*R*[*F* ^2^ > 2σ(*F* ^2^)] = 0.084
*wR*(*F* ^2^) = 0.230
*S* = 1.07
1283 reflections
83 parameters
1 restraint
H-atom parameters constrained
Δρ_max_ = 0.31 e Å^−3^
Δρ_min_ = −0.41 e Å^−3^

### 

Data collection: *CrystalClear* (Rigaku, 2005 ▶); cell refinement: *CrystalClear*; data reduction: *CrystalClear*; program(s) used to solve structure: *SHELXS97* (Sheldrick, 2008 ▶); program(s) used to refine structure: *SHELXL97* (Sheldrick, 2008 ▶); molecular graphics: *ORTEPIII* (Burnett & Johnson, 1996 ▶), *ORTEP-3 for Windows* (Farrugia, 1997 ▶) and *PLATON* (Spek, 2009 ▶); software used to prepare material for publication: *SHELXL97*.

## Supplementary Material

Crystal structure: contains datablock(s) I, global. DOI: 10.1107/S160053681103532X/jh2316sup1.cif

Structure factors: contains datablock(s) I. DOI: 10.1107/S160053681103532X/jh2316Isup2.hkl

Supplementary material file. DOI: 10.1107/S160053681103532X/jh2316Isup3.cml

Additional supplementary materials:  crystallographic information; 3D view; checkCIF report

## Figures and Tables

**Table 1:** Hydrogen-bond geometry (Å, °)

*D*—H⋯*A*	*D*—H	H⋯*A*	*D*⋯*A*	*D*—H⋯*A*
O2—H2⋯O1^i^	0.98	1.68	2.650 (2)	170

Symmetry code: (i) 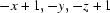.

**Fig. 1.**
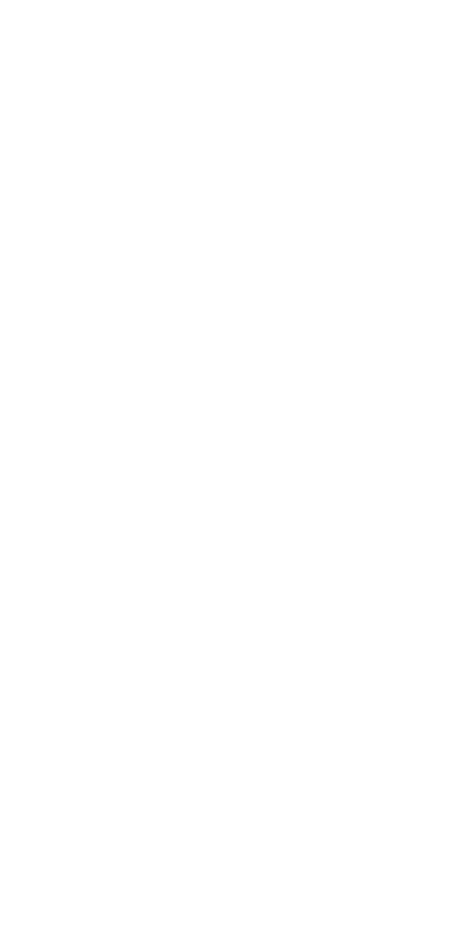
The molecular structure of the title compound with the atom-labelling scheme. Displacement ellipsoids are drawn at the 30% probability level. H atoms are represented as small spheres of arbitrary radii.

**Fig. 2.**
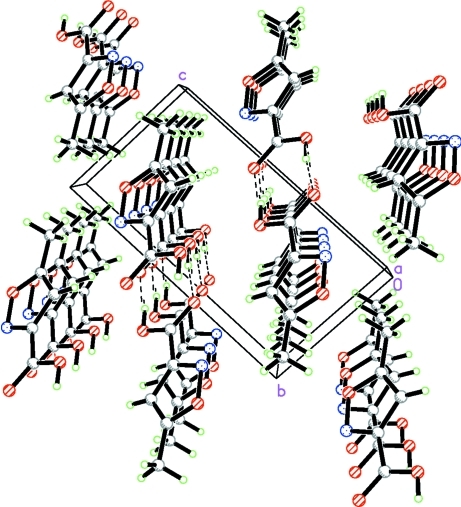
A packing view down the a axis showing the three dimensionnal network.Intermolecular hydrogen bonds are shown as dashed lines. H atoms have been omitted for the sake of clarity.

## supplementary crystallographic information

### Comment

5-Methylisoxazole-3-carboxylic acid is a potent inhibitor of the monoamine oxidase enzyme and excellent ligand for transition metals (Birk, *et al.*,2009) as well as other derivatives of isoxazole. As part of our interest in these compounds, we report here the crystal structure of the title compound.

The molecular structure of the title compound is shown in Fig. 1. All the non-H atoms of the title compound are located almost in one plane, as the atoms O1 and O2 are shifted just *ca* 0.0016Å out of the isoxazole ring plane.

The title compound formed dimer *via* intermolecular O—H···O hydrogen bonds and the dimers packed *via*π–π stacking interactions (3.234 Å).(Fig. 2).

### Experimental

The title compound was purchased commercially. Crystals suitable for X-ray diffraction were obtained by slow evaporation of an ethanol solution.

### Refinement

All H atoms attached to C atoms and O atoms were fixed geometrically and treated as riding with C—H = 0.93 Å (CH) or C—H = 0.96 Å and O—H = 0.9796 Å with *U*_iso_(H) = 1.2*U*_eq_(CH) and *U*_iso_(H) = 1.5*U*_eq_(O,CH_3_).

### Crystal data

**Table:**

C_5_H_5_NO_3_	*Z* = 2
*M**_r_* = 127.10	*F*(000) = 132
Triclinic, *P*1	*D*_x_ = 1.502 Mg m^−^^3^
Hall symbol: -P 1	Mo *K*α radiation, λ = 0.71073 Å
*a* = 4.9125 (10) Å	Cell parameters from 1283 reflections
*b* = 5.6909 (11) Å	θ = 3.7–27.5°
*c* = 10.464 (2) Å	µ = 0.13 mm^−^^1^
α = 82.21 (3)°	*T* = 293 K
β = 79.72 (3)°	Prism, colourless
γ = 78.96 (3)°	0.20 × 0.20 × 0.20 mm
*V* = 280.96 (10) Å^3^	

### Data collection

**Table:**

Rigaku SCXmini diffractometer	1283 independent reflections
Radiation source: fine-focus sealed tube	1052 reflections with *I* > 2σ(*I*)
graphite	*R*_int_ = 0.079
Detector resolution: 13.6612 pixels mm^-1^	θ_max_ = 27.5°, θ_min_ = 3.7°
CCD_Profile_fitting scans	*h* = −6→6
Absorption correction: multi-scan (*CrystalClear*; Rigaku, 2005)	*k* = −7→7
*T*_min_ = 0.975, *T*_max_ = 0.975	*l* = −13→13
2923 measured reflections	

### Refinement

**Table:**

Refinement on *F*^2^	Primary atom site location: structure-invariant direct methods
Least-squares matrix: full	Secondary atom site location: difference Fourier map
*R*[*F*^2^ > 2σ(*F*^2^)] = 0.084	Hydrogen site location: inferred from neighbouring sites
*wR*(*F*^2^) = 0.230	H-atom parameters constrained
*S* = 1.07	*w* = 1/[σ^2^(*F*_o_^2^) + (0.1395*P*)^2^] where *P* = (*F*_o_^2^ + 2*F*_c_^2^)/3
1283 reflections	(Δ/σ)_max_ < 0.001
83 parameters	Δρ_max_ = 0.31 e Å^−^^3^
1 restraint	Δρ_min_ = −0.41 e Å^−^^3^

### Special details

**Table:**

Geometry. All e.s.d.'s (except the e.s.d. in the dihedral angle between two l.s. planes) are estimated using the full covariance matrix. The cell e.s.d.'s are taken into account individually in the estimation of e.s.d.'s in distances, angles and torsion angles; correlations between e.s.d.'s in cell parameters are only used when they are defined by crystal symmetry. An approximate (isotropic) treatment of cell e.s.d.'s is used for estimating e.s.d.'s involving l.s. planes.
Refinement. Refinement of *F*^2^ against ALL reflections. The weighted *R*-factor *wR* and goodness of fit *S* are based on *F*^2^, conventional *R*-factors *R* are based on *F*, with *F* set to zero for negative *F*^2^. The threshold expression of *F*^2^ > σ(*F*^2^) is used only for calculating *R*-factors(gt) *etc*. and is not relevant to the choice of reflections for refinement. *R*-factors based on *F*^2^ are statistically about twice as large as those based on *F*, and *R*- factors based on ALL data will be even larger.

### Fractional atomic coordinates and isotropic or equivalent isotropic displacement parameters (Å^2^)

**Table:**

	*x*	*y*	*z*	*U*_iso_*/*U*_eq_	
O3	−0.2313 (3)	0.3175 (2)	0.15660 (15)	0.0539 (5)	
N1	−0.0692 (4)	0.1507 (3)	0.2343 (2)	0.0530 (6)	
C2	0.0502 (4)	0.2811 (3)	0.29544 (17)	0.0401 (5)	
C1	0.2432 (4)	0.1560 (3)	0.38652 (18)	0.0419 (5)	
C3	−0.0274 (4)	0.5290 (3)	0.26092 (19)	0.0436 (5)	
H3	0.0293	0.6551	0.2915	0.052*	
C4	−0.2024 (4)	0.5436 (3)	0.17357 (18)	0.0428 (5)	
O1	0.2945 (3)	−0.0687 (3)	0.40024 (16)	0.0560 (5)	
C5	−0.3597 (5)	0.7439 (4)	0.0956 (2)	0.0541 (6)	
H5A	−0.3371	0.7068	0.0071	0.081*	
H5B	−0.2886	0.8892	0.0971	0.081*	
H5C	−0.5553	0.7655	0.1323	0.081*	
O2	0.3450 (3)	0.2937 (2)	0.44412 (15)	0.0551 (5)	
H2	0.4849 (14)	0.1977 (10)	0.4948 (5)	0.083*	

### Atomic displacement parameters (Å^2^)

**Table:**

	*U*^11^	*U*^22^	*U*^33^	*U*^12^	*U*^13^	*U*^23^
O3	0.0681 (10)	0.0405 (9)	0.0617 (10)	−0.0087 (7)	−0.0357 (8)	−0.0030 (7)
N1	0.0638 (11)	0.0374 (10)	0.0650 (12)	−0.0080 (8)	−0.0332 (9)	−0.0017 (8)
C2	0.0447 (10)	0.0375 (10)	0.0398 (10)	−0.0074 (7)	−0.0116 (8)	−0.0032 (8)
C1	0.0446 (10)	0.0415 (10)	0.0408 (10)	−0.0082 (8)	−0.0103 (8)	−0.0028 (8)
C3	0.0496 (11)	0.0391 (11)	0.0455 (10)	−0.0088 (8)	−0.0139 (8)	−0.0058 (8)
C4	0.0495 (10)	0.0362 (10)	0.0446 (10)	−0.0066 (8)	−0.0137 (8)	−0.0036 (7)
O1	0.0646 (10)	0.0407 (9)	0.0655 (10)	−0.0029 (7)	−0.0291 (8)	0.0015 (7)
C5	0.0625 (13)	0.0463 (12)	0.0540 (12)	−0.0031 (9)	−0.0220 (10)	0.0009 (9)
O2	0.0624 (10)	0.0517 (10)	0.0581 (10)	−0.0081 (8)	−0.0295 (8)	−0.0057 (7)

### Geometric parameters (Å, °)

**Table:**

O3—C4	1.359 (2)	C3—C4	1.347 (3)
O3—N1	1.388 (2)	C3—H3	0.9300
N1—C2	1.317 (3)	C4—C5	1.482 (3)
C2—C3	1.404 (3)	C5—H5A	0.9600
C2—C1	1.481 (3)	C5—H5B	0.9600
C1—O1	1.249 (2)	C5—H5C	0.9600
C1—O2	1.270 (2)	O2—H2	0.9796
			
C4—O3—N1	109.46 (15)	C3—C4—O3	108.95 (18)
C2—N1—O3	104.75 (15)	C3—C4—C5	134.7 (2)
N1—C2—C3	112.28 (18)	O3—C4—C5	116.31 (18)
N1—C2—C1	118.65 (18)	C4—C5—H5A	109.6
C3—C2—C1	129.06 (18)	C4—C5—H5B	109.4
O1—C1—O2	125.6 (2)	H5A—C5—H5B	109.5
O1—C1—C2	119.38 (18)	C4—C5—H5C	109.5
O2—C1—C2	114.99 (17)	H5A—C5—H5C	109.5
C4—C3—C2	104.56 (17)	H5B—C5—H5C	109.5
C4—C3—H3	127.7	C1—O2—H2	109.6
C2—C3—H3	127.8		

### Hydrogen-bond geometry (Å, °)

**Table:**

*D*—H···*A*	*D*—H	H···*A*	*D*···*A*	*D*—H···*A*
O2—H2···O1^i^	0.98	1.68	2.650 (2)	170.

Symmetry codes: (i) −*x*+1, −*y*, −*z*+1.
